# Clinical Nurses as Second Victims After Patient Safety Incidents: A Meta‐Synthesis of Experiences, Coping and Support Needs

**DOI:** 10.1155/jonm/7053851

**Published:** 2026-07-30

**Authors:** Wei Zeng, Yuxin Yang, Li Zhang, Qingyun Li

**Affiliations:** ^1^ School of Nursing, Nanjing University of Chinese Medicine, Nanjing, China, njucm.edu.cn; ^2^ Department of Nursing, The Second Hospital of Nanjing, Affiliated to Nanjing University of Chinese Medicine, Nanjing, China, njmu.edu.cn

**Keywords:** meta-synthesis, nurse, patient safety, qualitative research, second victim

## Abstract

**Background:**

In patient safety incidents (PSIs), nurses often act as ‘second victims’, experiencing multifaceted distress, including psychological, physiological and professional burdens. In recent years, qualitative studies on nurses as second victims have increased; however, the evidence remains fragmented. Existing qualitative meta‐syntheses have limitations in terms of contextual diversity, coverage of incident types and exploration of support needs.

**Aim:**

To synthesise qualitative evidence on nurses as second victims after PSIs and provide implications for developing a multilevel support system.

**Methods:**

A qualitative meta‐synthesis approach was employed. Twelve Chinese and English databases were systematically searched from inception to 10 February 2025, with an updated search conducted on 14 October 2025. The 2020 Joanna Briggs Institute (JBI) critical appraisal checklist for qualitative research was used to appraise methodological quality. Data were synthesised using the two‐stage synthesis approach proposed by Sandelowski and Barroso.

**Results:**

A total of 21 qualitative studies involving 272 nurses across seven countries were included. Three synthesised themes were identified: (1) a state of disequilibrium under multiple shocks; (2) support systems undermined by blame and silence; and (3) reconstructing the self in the aftermath of trauma: multipath coping and recovery mechanisms.

**Conclusions:**

Nurses’ second‐victim experiences after PSIs are multidimensional, dynamic and shaped by incident severity, individual appraisal, organisational culture and accessibility of support. A just culture–based support system may help reduce blame and silence, promote psychological safety and facilitate recovery.

**Implications for Nursing Management:**

Nursing managers should recognise nurses’ postincident disequilibrium as a second‐victim response and embed structured support into PSI management. Strengthening just culture, psychological safety, peer support, confidential psychological care and protected recovery time may reduce blame, silence and help‐seeking barriers, thereby facilitating nurses’ recovery and safeguarding patient safety.

## 1. Introduction

Patient safety incidents (PSIs) represent systemic risks that cannot be entirely avoided in healthcare delivery. They refer to unintended events occurring during diagnosis and treatment that may have caused, or have already caused, patient harm, leading to prolonged hospitalisation, disability or even death [1]. The World Health Organization (WHO) has reported that more than 3 million deaths worldwide each year are attributable to PSIs [2]. Owing to their high incidence and severe consequences, PSIs have become a critical challenge that healthcare systems globally must urgently address.

Traditionally, efforts surrounding PSIs have primarily focused on patient remediation and root cause analysis, while the impact on healthcare professionals who are directly or indirectly involved has been relatively overlooked. In 2022, based on a synthesis of prior evidence, the European Researchers’ Network Working on Second Victims (ERNST) issued a consensus definition: ‘Any health care worker, directly or indirectly involved in an unanticipated adverse patient event, unintentional healthcare error, or patient injury and who becomes victimised in the sense that they are also negatively impacted’ [3]. As early as 2000, Wu first introduced the concept of the ‘second victim’ to describe the secondary psychological trauma experienced by healthcare professionals after medical errors [4]. In 2009, Scott et al. further defined the concept and developed a second‐victim recovery trajectory model, providing an important theoretical foundation for subsequent research [5].

The second‐victim phenomenon is highly prevalent among healthcare professionals and carries substantial potential harms. International studies suggest that approximately half of healthcare professionals experience similar trauma during their careers [6, 7]. A cross‐sectional survey in China involving 1194 nurses reported that 76.88% of participants had experienced a PSI [8]. PSIs may trigger multidimensional negative reactions among healthcare professionals, including anxiety, self‐doubt, insomnia and headaches [4, 9]. Such experiences can undermine professional identity and job satisfaction, increase the risks of absenteeism and burnout and may even contribute to subsequent PSIs, thereby creating a vicious cycle [10, 11]. Against the backdrop of a global nursing workforce shortage and persistently rising workloads, nurses’ physical and mental health directly affects the quality of nursing care and patient safety [12]. The theme of International Nurses Day 2025—‘Our Nurses. Our Future. Caring for nurses strengthens economies’—further underscores the importance of nurses’ health for the sustainability of health systems and socioeconomic stability [13].

Since Wu introduced the concept of the second victim, research on nurses as second victims in PSIs has steadily increased both in China and internationally. A growing body of qualitative studies across diverse contexts has examined nurses’ subjective experiences, coping strategies and support needs following PSIs. However, single qualitative studies are constrained by contextual or sampling limitations and therefore may not comprehensively capture the multidimensional predicament of nurses as second victims. Although existing qualitative meta‐syntheses have provided an important basis for understanding the second‐victim phenomenon among nurses, several limitations remain. First, some syntheses included only nurses from mainland China, resulting in a relatively limited geographical scope of evidence and insufficient representation of the complexity of nurses’ second‐victim experiences across different clinical contexts and healthcare environments. Second, some previous reviews mainly focused on nurses’ emotional experiences after PSIs, with limited attention to coping processes, support needs and factors that hinder access to support. Third, some reviews focused only on specific types of incidents, such as inpatient suicide, which limits the transferability of their findings to medication errors, device alarm‐related events and other types of PSIs [14–16].

Based on the above considerations, this study aimed to systematically synthesise qualitative studies on nurses as second victims in PSIs. Specifically, it sought to comprehensively examine nurses’ psychological, physiological and professional responses after such incidents and to clarify their coping pathways, support needs and the organisational–cultural factors influencing access to support. By integrating findings from studies conducted across different countries, clinical settings and incident types, this review aims to provide more comprehensive evidence for developing an actionable, multilevel and multistakeholder second‐victim support system.

## 2. Methods

### 2.1. Study Design

This study adopted a qualitative meta‐synthesis approach. Meta‐synthesis involves the systematic aggregation and reinterpretation of findings from multiple qualitative studies addressing the same topic through rigorous and transparent procedures, with the aim of generating higher‐level conceptual and theoretical understandings to inform future research and clinical practice [17, 18]. The study protocol was prospectively registered in PROSPERO (CRD420251002741) to ensure transparency and credibility of the research process. The reporting of this review followed the enhancing transparency in reporting the synthesis of qualitative research (ENTREQ) statement. This study was an evidence synthesis based on previously published qualitative studies and did not involve the recruitment of participants or the collection of new personal data; therefore, ethical approval was not required.

### 2.2. Search Strategy

The literature search was conducted in accordance with the Preferred Reporting Items for Systematic Reviews and Meta‐Analyses (PRISMA) guidelines [19], with appropriate adaptations to accommodate the characteristics of qualitative research. An initial exploratory search was undertaken in PubMed and CINAHL. Based on the research question, four core concepts were identified: patient safety, nurses, second victim, and qualitative studies. Controlled vocabulary terms and free‐text keywords specific to each database were combined using Boolean operators and truncation to construct the final search strategies. A comprehensive search was then performed across 12 Chinese and English databases: PubMed, Web of Science, Elsevier ScienceDirect, Embase, CINAHL, Cochrane Library, APA PsycNet and Scopus, as well as the China National Knowledge Infrastructure (CNKI), Wanfang Data, VIP Database and the Chinese Biomedical Literature Database (CBM). As this study aimed to synthesise published qualitative evidence rather than evaluate clinical trials, intervention effects, or ongoing trial projects, clinical trial registries were not included as primary search sources. The search covered the period from database inception to 10 February 2025. To ensure the currency of the evidence, an updated search was conducted on 14 October 2025 (Supporting File 1).

EndNote 21 was used for reference management, and duplicate records were removed through a combination of automated and manual checks. Two researchers independently screened titles, abstracts, and full texts according to the predefined inclusion and exclusion criteria. Any disagreements were resolved through discussion, with a third researcher consulted to reach a final consensus.

### 2.3. Inclusion and Exclusion Criteria

The inclusion and exclusion criteria were developed in accordance with the PICoS framework recommended by the Joanna Briggs Institute (JBI), as follows:

Population (P): Registered nurses working in frontline clinical settings; nursing managers and nursing students/interns were excluded.

Phenomena of interest (I): Nurses’ subjective experiences, support needs and coping strategies following PSIs.

Context (Co): PSIs occurring in hospitals of different levels and across various clinical departments.

Study design (S): Original studies employing qualitative research methods, including phenomenology, grounded theory, action research, narrative inquiry and other qualitative designs.

Exclusion criteria: publications in languages other than Chinese or English; studies for which full texts were unavailable or data were incomplete and could not be obtained after contacting the original authors; duplicate publications or studies with overlapping content; theses or dissertations, reviews, conference proceedings and case studies; mixed‐methods studies; and studies rated as Grade C based on the JBI quality appraisal.

Mixed‐methods studies were excluded because this review focused on synthesising findings generated from purely qualitative designs. The qualitative components of mixed‐methods studies often vary in reporting depth, analytic independence and extractability; therefore, excluding them helped maintain methodological consistency and avoid incorporating quantitatively derived findings into the thematic integration of qualitative evidence.

### 2.4. Quality Appraisal

The methodological quality of the included studies was appraised using the JBI critical appraisal checklist for qualitative research (2020 version) [20]. Two researchers who had received formal training in evidence‐based methodology independently conducted the appraisal. Studies were classified into three categories: Grade A (low risk of bias), indicating full compliance with the criteria; Grade B (moderate risk of bias), indicating partial compliance; and Grade C (high risk of bias), indicating noncompliance. Any discrepancies in appraisal results were resolved through discussion within the research team or with the involvement of a third reviewer. Only studies rated as Grade A or B were included, while Grade C studies were excluded to ensure the credibility and reliability of the synthesised findings.

### 2.5. Data Extraction

Data extraction was performed using the JBI Qualitative Assessment and Review Instrument (JBI‐QARI) data extraction tool [21]. Two researchers independently extracted data from the included studies. Extracted information comprised basic study characteristics, study aims and key findings relevant to nurses’ experiences as second victims in PSIs. Discrepancies between the two reviewers were resolved through discussion until consensus was reached within the research team.

### 2.6. Synthesis

Data analysis was conducted using the two‐stage qualitative evidence synthesis approach proposed by Sandelowski and Barroso [22].

The first stage was the meta‐summary, which aimed to systematically collate and descriptively aggregate the main findings of the included qualitative studies. Findings related to nurses’ subjective experiences, coping strategies and support needs as second victims in PSIs were extracted from each primary study. To reduce bias arising from differences in language, qualitative paradigms and national contexts, a standardised concept calibration process was applied. First‐order concepts, including participants’ quotations and experience‐near descriptions, and second‐order concepts, including authors’ themes and interpretations, were extracted separately. Because the research team conducted the synthesis primarily in Chinese, concepts and findings from English‐language studies were first translated into Chinese using professional translation software and then checked and refined by a bilingual researcher with reference to the original English wording and contextual meaning. During synthesis, concepts were compared based on conceptual equivalence rather than literal wording, with attention to their original methodological and contextual meanings. Similar concepts were merged only when their meanings were sufficiently comparable, whereas context‐specific concepts were retained for further comparison. Information on incident type, clinical department and participant characteristics was also retained during extraction and coding to avoid overgeneralising concepts beyond their original contexts. The final synthesised themes and representative concepts were translated into English for manuscript presentation and checked for consistency with the original sources. Disagreements were resolved through team discussion. The analytic process was guided by the thematic analysis framework developed by Braun and Clarke [23]. Through iterative coding, comparison and abstraction, initial concepts were generated. Subsequent studies were continuously compared with the emerging concepts, which were refined through supplementation or merging as needed, ultimately forming a structured set of themes and subthemes. For each theme, a frequency effect size was calculated as the number of studies containing the theme divided by the total number of included studies, thereby indicating the prevalence of each theme across studies and enhancing the transparency and richness of the qualitative synthesis [24]. The first author conducted the initial synthesis, and the second author reviewed both the analytic process and results. Consensus was reached before proceeding to the next stage.

The second stage was the meta‐synthesis, which involved interpretive integration of the themes generated in the meta‐summary. Through cross‐study comparison and triangulation of concepts, existing themes and subthemes were reorganised to develop a deeper, more comprehensive understanding of the phenomenon. The first and second authors independently conducted the meta‐synthesis, after which discrepancies were discussed and resolved. The third author reviewed the synthesised findings, and final agreement was achieved through discussion within the research team.

## 3. Results

**FIGURE 1:**
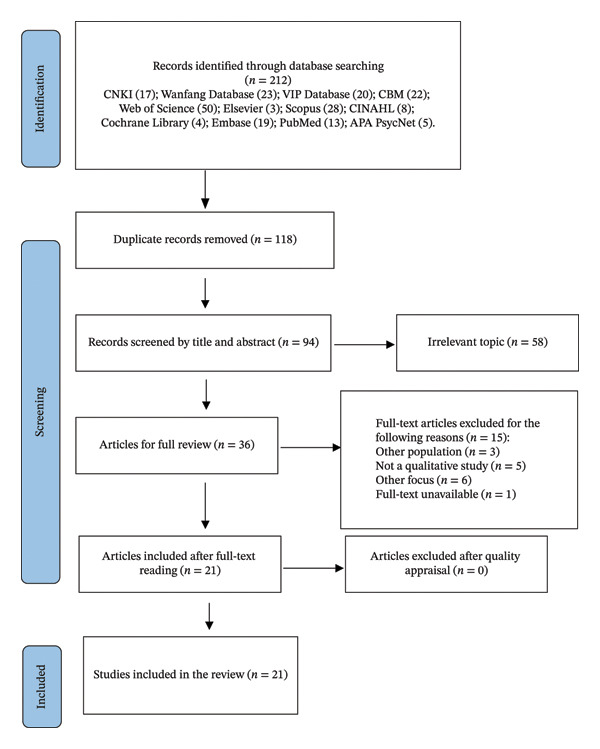
PRISMA flow diagram of study selection.

A total of 212 records were retrieved from 12 databases. After removing 118 duplicates, 94 records proceeded to title and abstract screening, of which 58 were excluded due to irrelevance to the review topic. Full texts of the remaining 36 records were assessed for eligibility, and 15 were excluded for not meeting the inclusion criteria. No studies were excluded due to a JBI quality appraisal rating of Grade C. Ultimately, 21 qualitative studies were included in the synthesis. Figure 1 presents the PRISMA flow diagram of the study selection process.

### 3.1. Characteristics of Included Studies

A total of 21 studies were included in this qualitative evidence synthesis, involving nurses from seven countries with diverse cultural backgrounds: 14 studies from China, two from Iran and one each from the United States, Singapore, Finland, Chile and South Africa. The included studies employed a range of qualitative designs: 12 used descriptive phenomenology, three used grounded theory, two were descriptive qualitative studies and one adopted a social constructionist perspective. Two studies did not report the specific qualitative design. Regarding data collection, semistructured interviews were the predominant method (*n* = 20); only one study combined in‐depth interviews with focus group discussions. Data analysis methods included Colaizzi’s seven‐step method (*n* = 9), thematic analysis (*n* = 5), content analysis (*n* = 3) and grounded theory analytic procedures (*n* = 3); one study did not clearly report its analytic method. Sample sizes ranged from 8 to 23 participants, comprising a total of 272 nurses. The basic characteristics of the included studies are presented in Table 1.

**TABLE 1:** Basic characteristics of the included studie**s**.

Included study	Country	Methodology	Aim	Data collection/analysis	Sampling/participants	Sample size (*n*)	Main findings (key themes)
M. Ajri et al., 2017 [25]	Iran	Unclear	To explain why emergency department nurses are at risk of making errors and the consequences of facing errors in the workplace	Semistructured interviews/content analysis	Purposive sampling/emergency department nurses from five teaching hospitals affiliated with Shahid Beheshti University of Medical Sciences	18	Psychological responses to errors; learning from errors; avoidant responses
R. Delacroix et al., 2017 [26]	USA	Phenomenology	To identify nurse practitioners’ (NPs’) behaviours, cognitions and coping mechanisms following medical errors	Semistructured interviews/thematic analysis	Convenience sampling/NPs from the Tampa Bay Advanced Practice Nurses Council	10	The paradox of error victimisation; primacy of responsibility and mindfulness; yearning for forgiveness and a supportive other; coping with a new reality is context dependent
Xu Jing et al., 2018 [27]	China	Phenomenology	Nurses’ experiences of becoming second victims in adverse medical events	Semistructured interviews/Colaizzi’s seven‐step method	Purposive sampling/nurses involved in adverse medical events from three tertiary (Grade A) hospitals	10	Stress symptom syndrome; helplessness; powerlessness; growth and gains
S. T. Chan et al., 2018 [28]	Singapore	Descriptive qualitative study	Nurses’ psychological responses, coping strategies and support needs as second victims after adverse events in Singapore	Semistructured interviews/thematic analysis	Purposive sampling/nurses from a large public acute‐care hospital in Singapore	8	Responding psychologically after the event; feeling others’ prejudice; having intrusive thoughts; drawing valuable lessons from the event; coping to recover after the event; taking responsibility for the mistakes made; finding self‐identity
Jin Yumei et al., 2019 [29]	China	Phenomenology	To explore paediatric nurses’ lived experiences as second victims in nursing adverse events and provide reference for subsequent organisational support programs	Semistructured interviews/unclear	Purposive sampling/registered nurses who had experienced nursing adverse events in the paediatric nursing unit of Ningbo Women and Children’s Hospital	17	Negative experiences of physical and psychological distress; lack of effective coping strategies during difficult times
Wang Ping et al., 2020 [30]	China	Phenomenology	To understand psychiatric nurses’ psychological experiences of becoming second victims in adverse events	Semistructured interviews/Colaizzi’s seven‐step method	Purposive sampling/psychiatric nurses who became second victims in adverse events at a tertiary Grade A hospital in Hangzhou	15	Experiencing psychophysiological trauma; seeking emotional support and professional guidance; posttraumatic growth
Chen Ying et al., 2021 [31]	China	Phenomenology	To explore the lived experiences of early‐career male nurses as second victims in nursing adverse events and to provide implications for clinical nursing managers to strengthen hospital safety management and stabilise the nursing workforce	Semistructured interviews/Colaizzi’s seven‐step method	Purposive sampling/early‐career male nurses employed in four general hospitals in Hangzhou	11	Commonly experiencing second‐victim syndrome; entering a context of blaming others and self‐blame; changes in professional cognition; adopting self‐protective measures; difficulty obtaining effective support and coping resources
Hao Lili et al., 2021 [32]	China	Unclear	To explore nurses’ psychological experiences and coping strategies as second victims in nursing adverse events and to inform the development of improved management strategies and greater attention to and support for second victims	Semistructured interviews/content analysis	Purposive sampling/nurses in four general hospitals in Shandong Province who had experienced nursing adverse events	10	Negative psychological experiences; process coping; growth and gains
Jia Yanan et al., 2021 [33]	China	Phenomenology	To understand emergency nurses’ psychological experiences of becoming second victims in nursing (safety) adverse events and to provide reference for nursing managers	Semistructured interviews/Colaizzi’s seven‐step method	Convenience sampling/emergency nurses involved in nursing safety incidents at The First Affiliated Hospital of Zhengzhou University	14	Psychological responses during the initial phase of the incident; psychological responses during the resolution phase; expectations as a second victim
Guan Ruixin et al., 2023 [34]	China	Grounded theory	To explore the trauma recovery process of second victims following ICU device alarm–related adverse events	Semistructured interviews/grounded theory analytic procedures	Purposive sampling/ICU nurses in a tertiary Grade A general hospital in Jilin Province	21	Appraisal phase; turbulence phase; recovery phase; control phase; continuation phase
Li Shanshan et al., 2023 [35]	China	Interpretive phenomenology	To explore psychological resuscitation pathways among clinical nurses who experienced patient safety incidents and to provide reference for developing supportive interventions	Semistructured interviews/Colaizzi’s seven‐step method	Purposive sampling/clinical nurses identified as having experienced PSIs in the adverse event reporting system of the Affiliated Hospital of Qingdao University	11	Psychological confusion stage; incident reaction stage; intrusive thinking stage; active introspection stage; emotional recovery stage; positive coping stage
Zhang Hong et al., 2023 [36]	China	Phenomenology	To understand second victims’ psychological experiences after obstetric nursing adverse events and to inform the development of targeted support systems	Semistructured interviews/Colaizzi’s seven‐step method	Purposive sampling/nurses in the obstetric unit of Huzhou Maternal and Child Health Care Hospital who experienced adverse events (Jan 2021–Dec 2022)	12	Changes in cognitive experiences; changes in coping experiences; support needs
Zhang Longbao et al., 2023 [37]	China	Phenomenology	To understand the lived experiences of early‐career paediatric nurses during adverse events and to provide evidence for nursing managers to develop clinical strategies	Semistructured interviews/Colaizzi’s seven‐step method	Purposive sampling/early‐career nurses who experienced adverse events in a tertiary Grade A children’s hospital in Suzhou	12	Bearing multiple pressures; receiving multidimensional support; rerceiving internal and external growth
Zhang Yuewei et al., 2024 [38]	China	Phenomenology	To gain an in‐depth understanding of psychological experiences and expectations of early‐career nurses in general ICUs as second victims after nursing adverse events and to inform nursing management practice	Semistructured interviews/Colaizzi’s seven‐step method	Unclear/15 early‐career ICU nurses who became second victims after adverse events in tertiary Grade A general hospitals across different regions of Shandong Province	15	Psychological stress during the initial phase of the incident; psychological stress during the resolution phase; expectations as a second victim
M. Kappes et al., 2024 [39]	Chile	Grounded theory	To describe the coping trajectory of ICU nurses in Chilean public hospitals as second victims	In‐depth interviews and focus groups/grounded theory analytic procedures	Theoretical sampling/ICU nurses working in Chilean public hospitals who had experienced an adverse event (AE) and became second victims	11	Perception of support when facing an AE; perception of helplessness when facing an AE; initiators of AE; responses when facing an AE; professional responsibility; perception of AE
S. Mahat et al., 2025 [40]	Finland	Descriptive qualitative study	To explore registered nurses’ psychological responses after medication errors, perceived support needs and expectations for support programs	Semistructured interviews/thematic analysis	Convenience sampling/registered nurses in Finland pursuing an advanced degree (Master’s or PhD) at a university	11	Contributing factors behind the second victim phenomenon; emotional responses of nurses after error; support received by nurses; the desired need for a support program for second victims
M. Mohsenpour et al., 2018 [41]	Iran	Phenomenology	To explore Iranian nurses’ psychological experiences as ‘at‐fault practitioners’ following nursing errors	Semistructured interviews/thematic analysis	Purposive sampling/nurses with clinical experience and experience of nursing errors from four public or private general and speciality hospitals in Tehran	8	Wandering in unpleasant feelings; wandering in the conscience court; being arrested in time; time for change; spiritual exercise
N. Zola et al., 2024 [42]	South Africa	Qualitative study informed by social constructionism	To explore nurses’ emotional well‐being and clinical practice following inpatient suicide in a psychiatric hospital	Semistructured interviews/thematic analysis	Purposive sampling/nurses who lost a patient to inpatient suicide while working at Weskoppies Psychiatric Hospital, South Africa	12	Emotional reactions; psychological effects; clinical practice factors; support systems; attitudes about suicide prevention and availability of approved protocols
Cao Na et al., 2025 [43]	China	Grounded theory	To explore the trauma recovery pathway of ICU nurses as second victims after PSIs and to inform supportive intervention strategies	Semistructured interviews/grounded theory analytic procedures	Purposive and theoretical sampling/nurses identified via the adverse event reporting system of a tertiary Grade A hospital in Zhengzhou who had experienced PSIs in the past three years	23	Incident response phase; stress phase; adjustment phase; return‐to‐work phase
Xie Xinxin et al., 2025 [44]	China	Phenomenology	To understand new nurses’ psychological experiences as second victims and to inform efforts to support their healthy development	Semistructured interviews/Colaizzi’s seven‐step method	Purposive sampling/unclear	8	Negative posttrauma experiences; positive recovery after trauma; posttrauma learning or growth
Qianfei Li et al., 2025 [45]	China	Descriptive qualitative study	From nurses’ perspectives, to identify and explore barriers and facilitators to second victims’ support‐seeking	Semistructured interviews/content analysis	Purposive sampling/nurses from a tertiary Grade A hospital in Shanxi Province, China	15	Individual factors; role conflict among managers; organisational support dilemmas; accessible support systems; need for diversified support

### 3.2. Results of Quality Appraisal

Quality appraisal indicated that all included studies met most items of the JBI critical appraisal checklist for qualitative research. Two studies were rated as Grade A and 19 as Grade B; no study was rated as Grade C. Detailed appraisal items and results are provided in Table 2.

**TABLE 2:** Quality appraisal using the Joanna Briggs Institute (JBI) critical appraisal checklist for qualitative research (Y = yes; N = no; U = not reported).

Included study	Q1	Q2	Q3	Q4	Q5	Q6	Q7	Q8	Q9	Q10	Overall appraisal
M. Ajri et al., 2017 [25]	U	Y	Y	U	Y	N	N	Y	Y	Y	B
R. Delacroix et al., 2017 [26]	Y	Y	Y	Y	Y	Y	Y	N	Y	Y	B
Xu Jing et al., 2018 [27]	Y	Y	Y	U	Y	N	N	Y	N	Y	B
S. T. Chan et al., 2018 [28]	Y	Y	Y	Y	Y	N	Y	Y	Y	Y	B
Jin Yumei et al., 2019 [29]	Y	Y	Y	U	Y	N	N	Y	Y	Y	B
Wang Ping et al., 2020 [30]	Y	Y	Y	U	Y	N	N	Y	N	Y	B
Chen Ying et al., 2021 [31]	Y	Y	Y	U	Y	N	N	Y	N	Y	B
Hao Lili et al., 2021 [32]	U	Y	Y	U	Y	N	N	Y	Y	Y	B
Jia Yanan et al., 2021 [33]	Y	Y	Y	U	Y	N	Y	Y	N	Y	B
Guan Ruixin et al., 2023 [34]	Y	Y	Y	U	Y	U	N	Y	Y	Y	B
Li Shanshan et al., 2023 [35]	Y	Y	Y	U	Y	N	N	Y	Y	Y	B
Zhang Hong et al., 2023 [36]	Y	Y	Y	U	Y	N	N	Y	Y	Y	B
Zhang Longbao et al., 2023 [37]	Y	Y	Y	U	Y	N	N	Y	N	Y	B
Included study	Q1	Q2	Q3	Q4	Q5	Q6	Q7	Q8	Q9	Q10	Overall appraisal
Zhang Yuewei et al., 2024 [38]	Y	Y	Y	U	Y	N	N	N	N	Y	B
M. Kappes et al., 2024 [39]	Y	Y	Y	Y	Y	N	Y	Y	Y	Y	B
S. Mahat et al., 2025 [40]	Y	Y	Y	Y	Y	Y	Y	Y	Y	Y	A
M. Mohsenpour et al., 2018 [41]	Y	Y	Y	Y	Y	N	Y	Y	Y	Y	B
N. Zola et al., 2024 [42]	Y	Y	Y	Y	Y	Y	Y	Y	Y	Y	A
Cao Na et al., 2025 [43]	Y	Y	Y	U	Y	N	N	Y	Y	Y	B
Xie Xinxin et al., 2025 [44]	Y	Y	Y	U	Y	N	N	Y	Y	Y	B
Qianfei Li et al., 2025 [45]	Y	Y	Y	Y	Y	N	N	N	Y	Y	B

*Note:* Q1: Is there congruity between the stated philosophical perspective and the research methodology? Q2: Is there congruity between the research methodology and the research question or objectives? Q3: Is there congruity between the research methodology and the methods used to collect data? Q4: Are the data adequately represented, and is the data analysis appropriate? Q5: Is there congruity between the research methodology and the interpretation of results? Q6: Is the researcher’s positionality (e.g., cultural background and values) clearly stated? Q7: Is the influence of the researcher on the research, and/or the influence of the research on the researcher, addressed? Q8: Are the participants and their perspectives adequately represented? Q9: Was ethical approval obtained, and if not reported, is there a rationale? Q10: Do the conclusions drawn in the research report flow logically from the analysis/interpretation of the data?

### 3.3. Synthesised Findings

A total of 21 qualitative studies were included in this review, covering nurses’ experiences as second victims in PSIs, including their experiences, coping strategies and support needs. Through meta‐summary of the first‐ and second‐order concepts, six themes were identified, with frequency effect sizes ranging from 52% to 100% (Supporting File 2). In this review, a higher frequency effect size indicates that the corresponding experience or support need was reported across a larger proportion of the included studies, suggesting greater recurrence and salience within the synthesised qualitative evidence. During the meta‐synthesis stage, these six themes were further integrated into three overarching themes: (1) a state of disequilibrium under multiple shocks; (2) support systems undermined by blame and silence; and (3) reconstructing the self in the aftermath of trauma: multipath coping and recovery mechanisms (Supporting File 3). The synthesised findings are presented in Table 3.

**TABLE 3:** Synthesised themes and subthemes.

Theme	Subthemes
A state of disequilibrium under multiple shocks	Disequilibrium in the emotional–cognitive system
	Somatic manifestations of the stress response
	Impaired professional identity

Support systems undermined by blame and silence	Caught between a desire for support and fear of blame
	The ‘invisible victim’ who is not seen

Reconstructing the self in the aftermath of trauma: multipath coping and recovery mechanisms	From self‐protection to active adjustment: individual coping to maintain functioning
	From coping alone to facing it together: relational and professional support as a critical turning point

#### 3.3.1. A State of Disequilibrium Under Multiple Shocks

##### 3.3.1.1. Disequilibrium in the Emotional–Cognitive System

Following PSIs, nurses commonly experience a state of ‘disequilibrium’ at both emotional and cognitive levels. Multiple studies have shown that the more severe the harm to the patient, the stronger nurses’ negative emotions—such as anxiety and self‐blame—become. Meanwhile, uncertainty regarding potential legal or professional accountability further intensifies this tension and unease [27, 34, 38, 41, 43, 44]. Some nurses reported that even though they had informed patients and families of risks in accordance with standard procedures, they still had to face the consequences of the incident and experienced a profound sense of grievance [33, 38].

Deep guilt and shame arising from highly internalised responsibility constituted another salient manifestation of emotional–cognitive disequilibrium. In several studies, nurses tended to attribute the incident to ‘errors in personal judgement’ or ‘insufficient professional competence’, even when they recognised the contribution of systemic factors such as excessive workload, equipment problems or process deficiencies. This strongly internalised sense of responsibility gradually evolved into persistent guilt and self‐reproach after the incident [26, 30, 31, 41]. Some nurses repeatedly revisited the course of events and became trapped in endless hypothetical reflections, resulting in sustained self‐doubt and diminished confidence [31, 32, 36–38, 43].

In addition, nurses endured emotional trauma closely tied to interpersonal relationships and professional evaluation. Nurses who had formed a deep emotional bond with patients felt sadness when the incident resulted in harm [38, 42, 43]. Others reported complex emotions in which anger and self‐blame were intertwined because they felt they had ‘failed to save the patient’ [42]. Multiple studies also indicated that nurses commonly worried about professional stigma after the event, fearing that they would be labelled as a ‘problem nurse’. Such concerns often extended into social interactions and contributed to pronounced social anxiety [28, 35, 36, 43–45].

“I asked the head nurse, ‘How is it going?’ She said, ‘Don’t worry about it—wait for the notice.’ At that moment, I felt it was over” [27].

“If I had been more careful, maybe the outcome wouldn’t have been like that” [38].

“If the patient didn’t wake up… my colleagues would label me, saying I’m just that kind of person” [28].

##### 3.3.1.2. Somatic Manifestations of the Stress Response

After experiencing a PSI, nurses exhibited marked physiological stress responses and changes in bodily functioning. At the time of the incident or in the immediate aftermath, some nurses reported tachycardia, rapid breathing and even transient mental blankness or confusion [31, 33, 35, 41, 44]. During critical moments of incident management, these symptoms interfered with nurses’ clinical judgement and ability to respond, making it difficult for them to regulate emotions promptly and return to a normal working state.

Following the acute reaction, sleep disturbance emerged as a common and persistent physiological response. Across multiple studies, nurses frequently reported difficulty falling asleep and frequent nighttime awakenings after the event [25, 26, 28, 30, 35, 42, 43]. In addition, some nurses experienced somatic symptoms characterised by appetite changes and weight loss, further increasing the physical and psychological burden at work [27, 30].

“I was stunned—I could only hear my heartbeat” [31].

“Those days I had a really poor appetite, and I had no energy at work” [27].

##### 3.3.1.3. Impaired Professional Identity

Adverse events made it difficult for nurses to derive the sense of value and accomplishment they previously obtained from their work; instead, they experienced low mood and occupational burnout. Some nurses reported that the incident made their work feel ‘meaningless’, fostering avoidance and passive coping tendencies. This was particularly evident among nurses in high‐intensity, high‐risk settings (e.g., ICU and psychiatric wards), who were more likely to consider transferring departments or leaving the profession [30, 38, 43]. Under the combined weight of guilt, self‐blame and external pressure, some nurses began to doubt their professional competence, with reduced job satisfaction and pessimistic expectations about their career prospects [26, 28, 30, 31, 41–43].

“After going through this… I felt my work had no meaning” [43].

“I’ve already considered changing careers—anyway, I can’t do this for long. I don’t want to take this kind of risk again” [31].

#### 3.3.2. Support Systems Undermined by Blame and Silence

##### 3.3.2.1. Caught Between a Desire for Support and Fear of Blame

After a PSI, nurses generally hoped to receive emotional understanding and support. Some nurses emphasised that understanding and trust from colleagues and supervisors—especially messages conveying ‘you can still do your job well in the future’—could effectively help them recover from negative emotions [27, 30, 33, 38, 44]; in addition, tolerance and forgiveness from patients and their families also played a pivotal role in alleviating guilt and self‐blame [28, 38].

However, blame‐ and punishment‐oriented approaches to incident handling substantially weakened nurses’ willingness to seek help. Although some organisations claim to promote a just culture, nurses often perceived it more as a slogan, with inadequate implementation in practice. Multiple studies indicated that nurses expected an open and fair institutional environment, believing that only within a ‘safe’ communication climate could they express the trauma brought about by the incident and proactively seek support [26, 40, 45]. Experiencing criticism or negative evaluations from supervisors after the incident often increased nurses’ psychological burden and discouraged them from seeking help from others [27, 40, 43, 45]. Caught in the tension between ‘wanting support’ and ‘fearing blame’, nurses often hovered at the margins of available support systems.

“The patient’s understanding and trust made me feel that I still had a chance to continue being a good nurse” [28].

“…the head nurse was very angry and had a negative attitude, which completely cut off my willingness to seek support” [40].

##### 3.3.2.2. The ‘Invisible Victim’ Who is Not Seen

Nurses’ psychological predicament as second victims after PSIs often lacked institutional attention and formal recognition. Some nurses reported that although they strongly desired professional support after the incident, corresponding resources were unavailable within the organisation [39, 40, 45].

Even when some support resources existed, they did not necessarily benefit every affected nurse. On the one hand, some nurses lacked awareness of the second‐victim concept and had limited self‐recognition when experiencing the impact of the incident [26, 27, 35, 44, 45]. On the other hand, nurses did not fully trust the professionalism and confidentiality of organisational support services [28, 42]. In addition, individual personality traits influenced the accessibility and uptake of support resources. Some nurses who were introverted or had strong self‐esteem tended to bear the burden alone [31, 39, 45].

“I realized my mindset had become very negative… I wanted to seek professional psychological counselling, but there was no dedicated psychological clinic” [45].

“For employee mental health services, I think most people are not really willing to go—mainly because they worry about confidentiality” [42].

“I’m the type who keeps things in—I’d rather endure it silently than say it out loud,” and “This is not a good thing—who would want to open up their ugly scars for others to see?” [45].

#### 3.3.3. Reconstructing the Self in the Aftermath of Trauma: Multipath Coping and Recovery Mechanisms

##### 3.3.3.1. From Self‐Protection to Active Adjustment: Individual Coping to Maintain Functioning

After a PSI, nurses adopt a range of individual‐level coping strategies to maintain basic work functioning and psychological balance. This process involves both avoiding and overdefending to insulate themselves from traumatic experiences and actively rebuilding a sense of safety and professional competence through self‐regulation and proactive learning.

Avoidant defensive coping is reflected in withdrawal from interpersonal interactions and high‐risk situations. Some nurses reduced social contact and avoided incident‐related contexts or procedures to distance themselves from work situations that felt ‘unsafe’ [30, 31]. After experiencing an error, some nurses tried to avoid performing similar nursing procedures, aiming to ‘do less and avoid high‐risk steps’, or engaged in excessive defensive behaviours to reduce the likelihood of making another mistake [25, 31, 33, 34, 36, 43].

In contrast, positive self‐regulation and reflective learning are manifested in nurses’ efforts to regulate emotions, strengthen adherence to procedural standards and engage in proactive learning to rebuild a sense of control over their work. Some studies reported that nurses encouraged themselves with an optimistic attitude or diverted attention to relieve anxiety and self‐blame [32, 40, 43]. Some nurses reinforced standardised double‐checking and self‐verification during procedures [25, 41, 43]. Others regarded the PSI as a ‘teachable moment’, actively consulting textbooks and relevant literature or seeking advice from more experienced colleagues to address gaps in knowledge and skills [25, 26, 28, 32, 35]. They also reflected on overlooked steps within existing workflows, shared lessons learnt with colleagues and achieved self‐improvement through externalising experience and promoting knowledge sharing [28, 35]. During this process, some nurses also reexamined the responsibilities and value of nursing work, recognised that nursing is closely related to patient safety and patients’ lives and deepened their understanding of professional responsibility and value through learning from the incident, strengthening risk awareness and improving professional competence [27, 41, 43].

“Ever since a patient pulled out the tracheal tube, I restrain patients regardless of whether they are conscious or unconscious—I’ve developed a psychological shadow” [43].

“…every year there are unplanned extubations; this won’t affect my attitude toward work in the future—if anything, it will help me accumulate more clinical experience” [32].

“I realized that nursing work in the ICU is very important—it is related to patients’ lives and safety” [43].

##### 3.3.3.2. From Coping Alone to Facing it Together: Relational and Professional Support as a Critical Turning Point

After a PSI, nurses do not always cope with and endure traumatic experiences in isolation. Emotional support within interpersonal relationships, together with institutional support at the organisational level, represents a critical condition that enables nurses to shift from ‘coping alone’ to ‘facing it together’.

Colleagues and supervisors were perceived as the most important and most immediate sources of workplace support. When a PSI occurred, experienced colleagues often proactively assisted the involved nurse in managing the incident, helping to alleviate feelings of isolation and helplessness [33, 37, 44, 45]. Some nurses actively confided in close colleagues to relieve stress [32, 44]. In addition, when nurse managers normalised ‘making mistakes’ as an occupational risk, emphasised learning from the incident and affirmed nurses’ engagement and effort, this played an important role in reducing shame and self‐blame [27, 36, 37].

Outside the workplace, family and faith provided nurses with important emotional support. Multiple studies noted that family is a key source of emotional support beyond the workplace and an essential resource for nurses coping with occupational trauma [35, 37, 40]. Some nurses also sought comfort through religion or spiritual beliefs to soothe inner unease [41].

Professional assistance, staffing arrangements and skills training constituted indispensable institutional safeguards. Nurses commonly reported the need for psychological support and adequate resources, viewing these as prerequisites for resuming safe practice after an incident. In some studies, nurses described marked stress reactions after managing adverse events and expressed the need for professional help to reduce psychological burden [42, 45]. In addition, nurses repeatedly emphasised the importance of time and space for recovery and adequate staffing, noting that understaffing and excessive workload not only increase the risk of errors but also reduce the possibility of rest and recovery afterward [33, 39, 43]. At the level of professional development, nurses also expressed needs for knowledge and skills training, hoping to enhance their observation and clinical judgement to prevent similar incidents at the source [30, 40, 42]. Through the combined effects of emotional and professional support, nurses were able to move from ‘individual coping’ to ‘facing it together’, gradually achieving postincident adjustment and recovery.

“…a senior colleague immediately taught me how to handle it and accompanied me to explain and apologize to the family” [37].

“The head nurse was really kind—she comforted me, saying everyone makes mistakes; just remember the lesson and don’t repeat it. I was deeply moved at that moment” [27].

“No matter what happens at work, my family will support me” [40].

“After the incident, I felt physically and mentally exhausted—how good it would have been if I could take leave and go out to rest” [43].

## 4. Discussion

Through a qualitative meta‐synthesis, this study portrays nurses’ post‐PSI ‘disequilibrium’ as second victims across multiple dimensions—emotional–cognitive responses, physiological reactions and occupational functioning—and further elucidates the profound influences of organisational culture, support resources and interpersonal relationships on their coping patterns and recovery trajectories. These findings provide important evidence for understanding the second‐victim phenomenon and for developing effective support systems.

### 4.1. Multiple Contributors to Nurses’ Post‐PSI ‘Disequilibrium’

Our findings indicate that nurses’ ‘disequilibrium’ after PSIs results from the combined effects of incident severity and uncertainty, negative feedback within the organisational environment and individuals’ internal cognitive appraisals.

The severity of the incident itself and the uncertainty embedded in the response process are key triggers of nurses’ disequilibrium. The synthesised evidence shows that the extent of patient harm is closely associated with the magnitude of nurses’ psychological impact; prior research has demonstrated that the duration of second‐victim symptoms increases with the degree of patient harm [46]. Building on this, uncertainty during incident management further amplifies the impact: Nurses must not only confront potentially serious adverse outcomes for patients but also cope with unclear accountability processes and potential legal and professional risks. When organisations fail to provide timely and transparent information feedback during incident handling, this ‘suspension’ of unknown consequences can substantially increase nurses’ psychological burden [47], leaving them in persistent tension and anxiety.

A blame‐oriented organisational culture and the absence of effective support systems can further intensify and prolong this disequilibrium. Evidence suggests that a just culture can effectively mitigate second victims’ distress responses [48]. However, the present synthesis indicates that nurses are often embedded in an ‘invisible’ blame culture and exposed only to formalistic support arrangements. Such a nonsupportive climate not only blocks avenues for emotional expression, but also—through the latent threat of labelling and professional stigmatisation—pushes nurses toward avoidant coping strategies. Caught between a desire for support and fear of blame, nurses’ psychological recovery is constrained by the external environment, making it difficult to regain stability in a timely manner.

In addition, under the backdrop of high societal expectations for the healthcare profession, nurses often develop a harsh self‐attribution style [49]. This synthesis shows that even when nurses recognise that adverse events are related to systemic factors such as heavy workload, understaffing and process deficiencies, they still tend to attribute responsibility to personal factors, such as ‘not being careful enough’ or ‘insufficient competence’. This highly internalised attribution of responsibility readily evokes deep guilt and shame, thereby transforming an initial stress response into self‐denial of one’s professional identity and value. Weiner’s attribution theory of responsibility suggests that attributing failure to internal, ability‐related causes can lead to strong self‐denial and doubt [50].

### 4.2. Systemic Harms of the ‘Disequilibrium’ State

Nurses’ post‐PSI ‘disequilibrium’ is not a transient emotional fluctuation but a multidimensional disruption involving psychological, physiological and occupational functioning. Its impact extends beyond individual well‐being to the stability of the nursing workforce and patient safety, posing harms that should not be overlooked.

First, this ‘disequilibrium’ seriously threatens nurses’ physical and mental health and may even progress to severe psychological disorders. The present synthesis shows that nurses commonly experience persistent emotional–cognitive distress after PSIs, including anxiety, guilt and self‐blame, accompanied by physiological symptoms such as insomnia and reduced appetite. Such mind–body interplay is not an isolated occurrence but rather a widely observed stress response [49]. If this psychophysiological dysregulation is not promptly recognised and addressed, it may readily evolve into prolonged psychological depletion and occupational burnout and may even trigger posttraumatic stress disorder (PTSD) [51], thereby substantially compromising nurses’ overall health.

Second, the ‘disequilibrium’ state undermines the stability of the nursing workforce. Recent evidence indicates that the severity of second‐victim distress is significantly associated with nurses’ turnover intention, absenteeism and work‐related quality of life [52]. Statements in the included studies such as ‘I’ve already considered changing careers’ and ‘I don’t want to take this kind of risk again’ further corroborate this linkage. Against the backdrop of a persistent global nursing workforce shortage [12], sustained neglect of second victims’ needs may accelerate the loss of frontline nurses, further increasing workload pressures and jeopardising the sustainability of nursing services.

More importantly, nurses’ ‘state of disequilibrium’ may further translate into patient safety risks. The findings of this review showed that, after an incident, some nurses adopted avoidant or overly defensive coping strategies to prevent making another mistake, such as deliberately avoiding high‐risk procedures, increasing unnecessary patient restraints or engaging in compulsive repeated checking. Although such fear‐driven ‘defensive clinical behaviours’ may appear to reflect caution, they may in fact divert nurses’ attention, reduce the quality of nursing care and even contribute to new PSIs through excessive anxiety [53]. Taken together, PSIs may not only cause short‐term individual harm to nurses but may also form a vicious cycle of ‘PSI occurrence ⟶ multidimensional emotional, physical and professional disequilibrium among nurses ⟶ defensive clinical behaviours or turnover intentions ⟶ increased patient safety risks’.

### 4.3. Building an ‘Effective’ Second‐Victim Support System

The synthesised findings suggest that nurses’ difficulty in accessing timely and effective support after PSIs cannot be attributed to a single factor. Rather, it is shaped by interacting organisational, individual and cultural–psychological mechanisms: (1) at the organisational level, the lack of systematic support resources and clear pathways often turns ‘needing support’ into ‘having nowhere to go’; (2) at the individual level, insufficient awareness of the concept and harms of second‐victim experiences makes it difficult for nurses to promptly recognise and acknowledge ‘I need help’; and (3) at the cultural–psychological level, a blame‐oriented and stigmatising climate renders help‐seeking a high‐risk choice. Therefore, building an effective support system requires coordinated efforts across organisational, individual and cultural–psychological dimensions.

At the organisational level, institutionalisation and process standardisation are needed to address the dilemma of ‘needs without access’. In this synthesis, many nurses reported that after an incident they ‘wanted professional help but there was no corresponding department’, or ‘wanted time off to recover but staffing was too tight’, reflecting gaps in organisational support resources. Organisational support theory posits that the higher the perceived organisational support, the stronger employees’ emotional recovery capacity in stressful situations and the lower their level of occupational burnout [54]. Accordingly, second‐victim support should be embedded within adverse event management procedures. After a PSI, designated personnel could conduct stress assessments for the involved nurse and, based on the results, provide tiered support options (e.g., referral to psychological counselling, arrangements for short‐term rest and recuperation). These procedures should be written into institutional policies and work manuals in the form of flowcharts or step‐by‐step guidance to increase the visibility and operability of support pathways. To enhance the practical relevance of management implications, the support process can be structured around nurses’ recovery trajectories as a set of continuous support points, including early emotional support, subsequent stress assessment and needs identification, supportive debriefing and resource referral during incident management and follow‐up and occupational adaptation support during the recovery stage. The specific timing of these support activities should be flexibly determined according to incident severity, nurses’ responses and institutional resources. In addition, information transparency constitutes an important form of organisational support [55]. Our synthesis indicates that informational opacity during incident handling increases nurses’ uncertainty and anxiety. It is therefore recommended that, while ensuring patient privacy and adherence to investigative protocols, departments or management units provide the involved nurse with timely, phased feedback on the progress and outcomes of investigations, along with explanations of the rationale for key decisions. This may help reduce fear of ‘unknown consequences’ and enhance nurses’ trust in organisational justice.

At the individual level, education and training should be strengthened to improve nurses’ awareness of second‐victim concepts and potential harms. This synthesis found that many nurses were unable to identify their own stress responses, struggled to recognise and acknowledge ‘I need help’, and even denied the legitimacy of receiving support because of intense shame. The help‐seeking process model conceptualises help‐seeking as a multistage process in which problem recognition is the primary prerequisite for initiating help‐seeking behaviour [56]. If nurses fail to recognise post‐PSI stress responses as a ‘health problem requiring intervention’, they are unlikely to progress to subsequent stages such as help‐seeking intention and action. In addition, this study found that nurses’ coping responses after PSIs did not follow a single pathway. This difference suggests that nurses’ coping process is not simply negative or positive but dynamically shifts among self‐protection, risk avoidance and meaning reconstruction. Defensive coping strategies, such as avoiding similar procedures and reducing communication, may help nurses temporarily reduce their fear of making another mistake and maintain a minimal sense of control at work. However, if sustained over time, these strategies may reinforce occupational avoidance and fear of high‐risk work, thereby affecting subsequent nursing quality. When nurses are able to reinterpret the incident through active learning, experience sharing and reflection on care processes, the traumatic experience may be transformed into enhanced risk awareness and professional growth. According to stress and coping theory, the way individuals respond to stressful events is closely related to their cognitive appraisal of the event [57]. If nurses lack an accurate understanding of the second‐victim phenomenon, they may easily attribute PSIs to personal failure or incompetence, thereby reinforcing shame, self‐blame and defensive coping. Conversely, if nurses recognise that negative reactions after such incidents are common second‐victim responses and understand that these reactions are relatively common and amenable to intervention, they may be more likely to reappraise the incident as a professional situation that can be learnt from and improved. Therefore, second‐victim–related content should be systematically incorporated into patient safety education, preemployment training and continuing education, including typical symptoms, potential consequences and available support resources, so that nurses can move toward conscious help‐seeking and effective reflection.

At the cultural and psychological level, it is essential to reduce the ‘risk’ associated with seeking support and to change the situation where ‘resources exist but people are unwilling to use them’. The synthesised findings of this review showed that nurses’ silence and self‐management after PSIs were not merely attributable to insufficient support resources but were also related to inadequate implementation of just culture, stigma associated with help‐seeking and the ‘invisibility’ of second victims. When incident management remains centred on accountability and performance appraisal, nurses may interpret help‐seeking as a sign of ‘incompetence’ or ‘psychological weakness’, thereby suppressing emotional expression and support‐seeking. Vogel et al.‘s theory of the self‐stigma of seeking help suggests that when individuals anticipate negative self‐ or social evaluation associated with help‐seeking, they may perceive it as a ‘high‐risk behaviour’ and actively inhibit it [58]. Because Chinese studies accounted for a relatively large proportion of the included literature, some nurses’ self‐blame, shame and reluctance to seek help may be related to a cultural context that emphasises professional reputation, interpersonal evaluation and ‘face’. This cultural context may, to some extent, intensify nurses’ internalisation of responsibility. At the same time, although some non‐Chinese studies also reported guilt, shame and impaired professional confidence, their narratives additionally involved institutional factors, such as postincident accountability risks, the clarity of support procedures and the availability of organisational support. For example, Mahat et al.‘s study of Finnish registered nurses after medication errors reported nurses’ concerns about possible legal action, while Zola et al.‘s South African study involved the availability of support systems and relevant protocols [40, 42]. It should be noted that this review did not conduct a formal cross‐cultural stratified synthesis; therefore, the above points are intended as contextual interpretations rather than definitive cultural comparative conclusions. Thus, the core of effective support lies not only in ‘providing resources’ but also in reshaping a psychologically safe environment in which nurses feel able to express distress, receive understanding and avoid secondary harm. This safe environment depends not only on organisational cultural change but also on supportive responses from key interpersonal relationships, including colleagues, supervisors and family members, after the incident. Social support theory suggests that emotional support, informational support and practical assistance from others can reduce the adverse effects of stressful events on individuals’ physical and psychological health [59]. From the perspective of social support theory, different sources of support play distinct roles in nurses’ recovery. Listening, companionship and experience sharing from colleagues mainly provide emotional support, helping to reduce nurses’ sense of isolation and shame. Event management advice and timely feedback from senior nurses and nurse managers represent informational and appraisal support, which can help nurses understand the incident, regain a sense of control and experience the incident management process as learning rather than blame. Family support provides an emotional outlet and practical support relatively separate from workplace evaluation systems, thereby buffering the impact of professional appraisal pressure on nurses’ psychological well‐being. Therefore, hospitals and clinical departments should continue to promote a transition from a punitive orientation to a learning‐oriented just culture [60]. In incident discussions, problems should not be simplified as ‘who made the mistake’; instead, analyses should focus on system deficiencies, working conditions and process vulnerabilities, with learning from the incident clearly established as the primary goal. In performance appraisal and promotion processes, the occurrence of an adverse event should not be rigidly treated as a negative label. By sharing positive examples of help‐seeking and recovery, nurses can be guided to view seeking support as an expression of professional competence in protecting themselves and safeguarding patient safety, rather than as a sign of incompetence or vulnerability. In addition, the support system should not be limited to referral for psychological counselling but should also include peer support training, nonjudgemental communication training for nurse managers and guidance on necessary family support, so that nurses can move from ‘bearing the burden alone’ to ‘facing it together’.

### 4.4. Strengths and Limitations

This study followed the ENTREQ reporting guidelines and adopted Sandelowski and Barroso’s two‐stage synthesis approach, thereby enhancing the transparency and credibility of the findings. The results provide a theoretical basis for developing and optimising support systems for second victims. However, several limitations should be acknowledged. First, although studies from multiple countries were included, the sample was still predominantly composed of Chinese nurses, which may affect the applicability of the findings to other national contexts. Second, although the included studies covered different countries, clinical departments and incident types, subgroup meta‐synthesis was not feasible because studies within each potential stratum were few, unevenly distributed and methodologically heterogeneous. This may have weakened the ability to identify incident‐specific differences in nurses’ second‐victim experiences. Third, the qualitative components of mixed‐methods studies were not systematically included, which may have limited the breadth of the evidence base. Finally, the study population was restricted to frontline registered nurses, excluding nurse managers, nursing students and other relevant groups; therefore, the findings may not fully capture perspectives from management roles or different stages of professional development.

## 5. Conclusion

This meta‐synthesis of 21 qualitative studies suggests that nurses’ experiences as second victims after PSIs are multidimensional and may be shaped by multiple factors, including incident characteristics, individual cognition and organisational culture. Therefore, it is necessary to develop an effective second‐victim support system through coordinated efforts involving organisational policies, cultural climate and education and training. Future research should include nurses from more diverse countries and regions to compare the commonalities and differences in second‐victim experiences and support needs across different healthcare systems and cultural contexts. Future qualitative syntheses with stratified designs by country, clinical setting or incident type are recommended to reduce methodological limitations and better examine context‐specific differences. In addition, high‐quality mixed‐methods studies with independently extractable qualitative findings should be incorporated where appropriate. Future studies should also include nurse managers, nursing students and other relevant groups to explore organisational constraints from a management perspective and the unique experiences of nurses at different stages of professional development.

## Author Contributions

Wei Zeng: conceptualisation, methodology, data extraction, formal analysis and writing–original draft. Yuxin Yang: literature screening, quality appraisal, data extraction and writing–review and editing. Li Zhang: supervision, methodology, validation and writing–review and editing. Qingyun Li: validation, interpretation of findings and writing–review and editing.

## Funding

This study was supported by funds from Nanjing Medical Science and Technique Development Foundation (no. QRX17070 and GAX21281).

## Disclosure

All authors read and approved the final manuscript.

## Conflicts of Interest

The authors declare no conflicts of interest.

## Supporting Information

Additional supporting information can be found online in the Supporting Information section.

## Supporting information


**Supporting Information 1** Supporting File 1: Search strategy of the databases.

**Supporting Information 2** Supporting File 2: Derivation of meta‐summarised themes and subthemes from the concepts of the included studies.

**Supporting Information 3** Supporting File 3: Derivation of meta‐synthesised themes from meta‐summarised themes and subthemes.

## Data Availability

All data analysed in this review were extracted from previously published studies. The data supporting the findings of this study are available within the article and its supporting files.
