# Supplementary material for: Clinical Nurses as Second Victims After Patient Safety Incidents: A Meta‐Synthesis of Experiences, Coping and Support Needs
Source: J Nurs Manag. 2026 Jul 30;2026:7053851. doi: 10.1155/jonm/7053851 (PMC13424605; doi:10.1155/jonm/7053851)
Supplement: Supplementary file 1 — Supporting Information 1 Supporting File 1: Search strategy of the databases. [file JONM-2026-7053851-s001.docx]

| **Supplementary File 1: Search strategy of the databases** | |
| --- | --- |
| Database | Search strategy |
| CNKI | (Subject: Nurse + Nursing) AND (Keywords: Medical Event + Adverse Event + Nursing Error + Patient Safety (Fuzzy)) AND (Title/Abstract: Second Victim (Fuzzy)) AND (Keywords: Qualitative Research + Qualitative Study + Grounded Theory + Phenomenology (Fuzzy)) |
| Wanfang Database | Subject: (Nurse OR Nursing) AND Title or Keywords: (Medical Event OR Adverse Event OR Nursing Error OR Patient Safety) AND Full Text: (Second Victim) AND Title or Keywords:(Qualitative Research OR Qualitative Study OR Grounded Theory OR Phenomenology) |
| VIP Database | Title or Keywords = (Nurse OR Nursing) AND Title or Keywords = (Medical Event OR Adverse Event OR Nursing Error OR Patient Safety) AND Any Field = (Second Victim) AND Title or Keywords = (Qualitative Research OR Qualitative Study OR Grounded Theory OR Phenomenology) |
| CBM | (“Nurse”[Common Fields: Intelligent] OR “Nursing”[Common Fields: Intelligent]) AND (“Medical Event”[Common Fields: Intelligent] OR “Adverse Event”[Common Fields: Intelligent] OR “Nursing Error”[Common Fields: Intelligent] OR “Patient Safety”[Common Fields: Intelligent]) AND “Second Victim”[Common Fields: Intelligent] AND (“Qualitative Research”[Common Fields: Intelligent] OR “Qualitative Study”[Common Fields: Intelligent] OR “Grounded Theory”[Common Fields: Intelligent] OR “Phenomenology”[Common Fields: Intelligent]) |
| Web of science | (((TS=(nurses* OR nurse clinicians)) AND TS=(medical errors OR patient safety* OR medical mistake OR adverse event OR nursing error)) AND TS=(crime victims OR second victim)) AND TS=(qualitative research* OR hermeneutics OR ground theory OR Phenomenology) |
| Elsevier ScienceDirect | Title, abstract, keywords:(nurses) AND (medical errors OR patient safety OR nursing error OR adverse event ) AND (crime victims OR second victim) AND (qualitative research OR hermeneutics) |
| Scopus | Title, abstract, keywords：(nurses* OR “nurse clinicians”) AND（“medical errors” OR “patient safety*” OR “medical mistake” OR “adverse event” OR “nursing error” ）AND（“crime victims” OR “second victim”）AND（“qualitative research*” OR hermeneutics OR “ground theory” OR Phenomenology) |
| CINAHL | (All Fields: “Nurse” OR “Nursing”) AND (“Medical Event” OR “Adverse Event” OR “Nursing Error” OR “Patient Safety”) AND (“Qualitative Research” OR “Qualitative Study” OR “Grounded Theory” OR “Phenomenology”) |
| Cochrane Library | Date Run: 14/10/2025 Comment:  ID Search Hits #1 MeSH descriptor: [Nursing] explode all trees 4427 #2 MeSH descriptor: [Nurse Clinicians] explode all trees 185 #3 #1 OR #2 4522 #4 MeSH descriptor: [Medical Errors] explode all trees 4074 #5 MeSH descriptor: [Patient Safety] explode all trees 1054 #6 “adverse event” OR “nursing error” 35148 #7 #4 OR #5 OR #6 40076 #8 MeSH descriptor: [Qualitative Research] explode all trees 2782 #9 MeSH descriptor: [Hermeneutics] explode all trees 4 #10 “ground theory” OR Phenomenology 276 #11 #8 OR #9 OR #10 3033 #12 #3 AND #7 AND #11 4 |
| EMBASE | #1 ：'nurses'/exp OR nurses #2 ：'clinical nurse specialist' #3 ：#1 OR #2 #4 ：'medical error' #5 ：'patient safety' #6 ：'adverse event' #7 ：'nursing error' #8 ：#4 OR #5 OR #6 OR #7 #9 ：'crime victim' #10 ：'second victim' #11 ：#9 OR #10 #12 ：'qualitative research' #13 ：'hermeneutics' #14 ：'ground theory' #15 ：'phenomenology' #16 ：#12 OR #13 OR #14 OR #15 #17 ：#3 AND #8 AND #11 AND #16 |
| PubMed | #1："nurses*"[MeSH Terms] OR "nurse clinicians"[MeSH Terms]  #2："medical errors"[MeSH Terms] OR "patient safety*"[MeSH Terms] OR "medical mistake"[Title/Abstract] OR "adverse event"[Title/Abstract] OR "nursing error"[Title/Abstract]  #3："crime victims"[MeSH Terms] OR "second victim"[Title/Abstract]  #4："qualitative research*"[MeSH Terms] OR "hermeneutics"[MeSH Terms] OR "ground theory"[Title/Abstract] OR "Phenomenology"[Title/Abstract]  #5：#1 AND #2 AND #3 AND #4 |
| APA PsycNet | nurses* OR “nurse clinicians” AND Any Field: “medical errors” OR “patient safety*” OR “medical mistake” OR “adverse event” OR “nursing error” AND Any Field: “crime victims” OR “second victim” AND Any Field: “qualitative research*” OR hermeneutics OR “ground theory” OR Phenomenology |
