# Supplementary material for: Clinical Nurses as Second Victims After Patient Safety Incidents: A Meta‐Synthesis of Experiences, Coping and Support Needs
Source: J Nurs Manag. 2026 Jul 30;2026:7053851. doi: 10.1155/jonm/7053851 (PMC13424605; doi:10.1155/jonm/7053851)
Supplement: Supplementary file 2 — Supporting Information 2 Supporting File 2: Derivation of meta‐summarised themes and subthemes from the concepts of the included studies. [file JONM-2026-7053851-s002.docx]

| **Supplementary File 2: Derivation of meta-summarised themes and subthemes from the concepts of the included studies** | | |
| --- | --- | --- |
| Concepts from the included studies | Meta-summarised results | |
|  | Themes | Sub-themes |
| Anxiety triggered by incident uncertainty | Psychological responses (frequency effect size: 100%) | Anxiety and helplessness in uncontrollable situations Self-blaming internal depletion Emotional trauma and relational strain |
| Affective grief for the patient |  |  |
| Anger and frustration over being unable to save the patient |  |  |
| Shame associated with making an error |  |  |
| Profound guilt |  |  |
| Helplessness in being unable to influence outcomes |  |  |
| Social anxiety driven by professional stigma |  |  |
| Distress from repetitive “what-if” rumination |  |  |
| A sense of grievance over being unable to prevent the incident |  |  |
| Insomnia triggered by event “flashbacks” | Physiological responses (frequency effect size: 76%) | Functional bodily changes Stress-related physiological reactions |
| Acute autonomic responses to stress |  |  |
| Sleep disturbance caused by ruminative thinking |  |  |
| Sleep disturbance due to hypervigilance |  |  |
| Somatic manifestations of stress |  |  |
| Decreased appetite and weight loss |  |  |
| Reduced sense of professional value | Occupational impacts (frequency effect size: 76%) | Impaired professional identity Reduced work engagement Strengthened sense of responsibility |
| Declining enthusiasm for work |  |  |
| Recognising the importance of nursing work |  |  |
| Questioning one’s original career choice |  |  |
| Doubting one’s professional competence |  |  |
| Fear of work/work-related tasks |  |  |
| Learning and improvement from errors | Coping strategies (frequency effect size: 100%) | Active reflection and learning Positive self-adjustment Avoidant defensive coping Seeking help from others |
| Strengthening standardised practice |  |  |
| Engaging in excessive defensive behaviours |  |  |
| Timely remediation to control incident progression |  |  |
| Positive self-regulation |  |  |
| Seeking support from others |  |  |
| Avoiding similar work environments |  |  |
| Avoidant defensive reactions |  |  |
| Professional psychological assistance | Support needs (frequency effect size: 86%) | Psychological and emotional support Resource provision for recovery and growth A supportive institutional environment |
| Space/time for psychophysiological recovery after the event |  |  |
| Human resource support |  |  |
| Forgiveness and understanding from others |  |  |
| A just and psychologically safe communication climate |  |  |
| Channels for emotional expression and ventilation |  |  |
| Improving existing systems and procedures |  |  |
| Knowledge and skills training |  |  |
| Emotional comfort from the organisation and society |  |  |
| Non-punitive management approaches |  |  |
| Inappropriate handling approaches | Barriers to effective support (frequency effect size: 52%) | Absence of organisational support Constraints related to individual differences Lack of trust and psychological safety Insufficient awareness/knowledge |
| Fear of discrimination from others |  |  |
| Lack of trust in organisational support |  |  |
| Shame associated with making an error |  |  |
| Individual personality differences |  |  |
| Inadequate implementation of just culture |  |  |
| Limited understanding of the second-victim concept |  |  |
| Lack of institutionalised support |  |  |
