# Supplementary material for: Clinical Nurses as Second Victims After Patient Safety Incidents: A Meta‐Synthesis of Experiences, Coping and Support Needs
Source: J Nurs Manag. 2026 Jul 30;2026:7053851. doi: 10.1155/jonm/7053851 (PMC13424605; doi:10.1155/jonm/7053851)
Supplement: Supplementary file 3 — Supporting Information 3 Supporting File 3: Derivation of meta‐synthesised themes from meta‐summarised themes and subthemes. [file JONM-2026-7053851-s003.docx]

| **Supplementary File 3: Derivation of meta-synthesised themes from meta-summarised themes and sub-themes** | | | |
| --- | --- | --- | --- |
| Meta-synthesised results | | Meta-summarised results | |
| Themes | Sub-themes | Themes | Sub-themes |
| A State of Disequilibrium Under Multiple Shocks | Disequilibrium in the emotional–cognitive system | Anxiety and helplessness in uncontrollable situations | Psychological responses |
|  |  | Self-blaming internal depletion | Psychological responses |
|  |  | Emotional trauma and relational strain | Psychological responses |
|  | Somatic manifestations of the stress response | Functional bodily changes | Physiological responses |
|  |  | Stress-related physiological reactions | Physiological responses |
|  | Impaired professional identity | Impaired professional identity | Occupational impacts |
|  |  | Reduced work engagement | Occupational impacts |
| Support Systems Undermined by Blame and Silence | Caught between a desire for support and fear of blame | Psychological and emotional support | Support needs |
|  |  | Lack of trust and psychological safety | Barriers to effective support |
|  | The “invisible victim” who is not seen | A supportive institutional environment | Support needs |
|  |  | Absence of organisational support | Barriers to effective support |
|  |  | Insufficient awareness/knowledge | Barriers to effective support |
|  |  | Constraints related to individual differences | Barriers to effective support |
| Reconstructing the Self in the Aftermath of Trauma: Multi-path Coping and Recovery Mechanisms | From self-protection to active adjustment: individual coping to maintain functioning | Avoidant defensive coping | Coping strategies |
|  |  | Reduced work engagement | Occupational impacts |
|  |  | Positive self-adjustment | Coping strategies |
|  |  | Active reflection and learning | Coping strategies |
|  |  | Strengthened sense of responsibility | Occupational impacts |
|  | From coping alone to facing it together: relational and professional support as a critical turning point | Seeking help from others | Coping strategies |
|  |  | Resource provision for recovery and growth | Support needs |
